# Promoting the Differentiation of Neural Progenitor Cells into Oligodendrocytes through the Induction of Olig2 Expression: A Transcriptomic Study Using RNA-seq Analysis

**DOI:** 10.3390/cells12091252

**Published:** 2023-04-26

**Authors:** Katarzyna Pieczonka, Mohamad Khazaei, Michael G. Fehlings

**Affiliations:** 1 Division of Genetics and Development, Krembil Research Institute, University Health Network, Toronto, ON M5T 0S8, Canada; 2 Institute of Medical Sciences, University of Toronto, Toronto, ON M5S 1A8, Canada; 3 Division of Neurosurgery, Department of Surgery, University of Toronto, Toronto, ON M5T 1P5, Canada

**Keywords:** neural progenitor cells, oligodendrocytes, oligodendrocyte-related neurological diseases, transcription factor induction, tet-ON promoters, doxycycline, Olig2, RNA-seq, gene ontology, myelin sheath, axon conduction, regenerative medicine, small molecules, differentiation

## Abstract

Oligodendrocytes are the myelinating cells of the central nervous system that facilitate efficient signal transduction. The loss of these cells and the associated myelin sheath can lead to profound functional deficits. Moreover, oligodendrocytes also play key roles in mediating glial-neuronal interactions, which further speaks to their importance in health and disease. Neural progenitor cells (NPCs) are a promising source of cells for the treatment of oligodendrocyte-related neurological diseases due to their ability to differentiate into a variety of cell types, including oligodendrocytes. However, the efficiency of oligodendrocyte differentiation is often low. In this study, we induced the expression of the Olig2 transcription factor in tripotent NPCs using a doxycycline-inducible promoter, such that the extent of oligodendrocyte differentiation could be carefully regulated. We characterized the differentiation profile and the transcriptome of these inducible oligodendrogenic NPCs (ioNPCs) using a combination of qRT-PCR, immunocytochemistry and RNA sequencing with gene ontology (GO) and gene set enrichment analysis (GSEA). Our results show that the ioNPCs differentiated into a significantly greater proportion of oligodendrocytes than the NPCs. The induction of Olig2 expression was also associated with the upregulation of genes involved in oligodendrocyte development and function, as well as the downregulation of genes involved in other cell lineages. The GO and GSEA analyses further corroborated the oligodendrocyte specification of the ioNPCs.

## 1. Introduction

Oligodendrocytes are a type of glial cell that reside in the central nervous system (CNS) and are essential for the formation and maintenance of the myelin sheath, which facilitates efficient signal conduction throughout the nervous system by insulating axons. Furthermore, oligodendrocytes play an integral role in modulating glial–neuronal interactions, which further emphasizes their importance in health and disease. For example, oligodendrocytes play a role in regulating the release and clearance of neurotransmitters in the synapse, which is essential in order to maintain a delicate balance of neurotransmitter levels that can support proper neuronal signaling [1,2,3]. Additionally, oligodendrocytes also play a role in the immune response through the production and release of cytokines and other signaling molecules that are involved in the activation and recruitment of immune cells to sites of injury or infection [1]. However, several disorders and injuries, including multiple sclerosis, leukodystrophies as well as traumatic brain and spinal cord injuries, are characterized by the loss of oligodendrocytes and/or the failure of their function, thus leading to impaired axon conduction and neurological deficits [4]. Neural progenitor cells (NPCs) are a promising source of cells for the treatment of oligodendrocyte-related neurological diseases due to their ability to differentiate into oligodendrocytes, in addition to neurons and astrocytes. However, the differentiation of NPCs into oligodendrocytes is often inefficient, whereby the majority of cells tend to differentiate into astrocytes [5]. Therefore, additional efforts are required to promote the oligodendrogenic fate of NPCs to maximize their potential for use in demyelinating disorders and injuries.

In this regard, we previously developed a small-molecule treatment protocol in order to bias NPCs along the oligodendrogenic lineage while maintaining the tri-lineage differentiation potential of the cells [6]. The protocol aimed to mimic oligodendrocyte generation during development. Specifically, NPCs were patterned toward a caudal and ventral identity with retinoic acid and a sonic hedgehog agonist, respectively. Next, the cells were treated with platelet-derived growth factor (PDGF) and thyroxine for 14 days, leading to a population of cells that were oligodendrogenically biased (oNPCs). Importantly, the oNPCs remained tripotent and gave rise to a greater proportion of oligodendrocytes both in vitro and in vivo than untreated NPCs, and they additionally promoted remyelination, axonal sparing and behavioral improvements in a model of thoracic spinal cord injury [7,8].

However, this biasing method introduces challenges related to scalability, efficiency, and GMP-grade manufacturing due to the long protocol and the requirement for several small molecules. Moreover, it does not account for patients who experience variable degrees of injury and extents of demyelination. In order to improve scalability and to treat these variable patient presentations, we aimed to develop a single adjustable oligodendrogenic cell source that could be suitable for all patients by engineering NPCs to express Olig2 under the control of the doxycycline-inducible tet-ON system. Olig2 is a basic helix–loop–helix transcription factor that is involved in the early specification of NPCs into oligodendrocyte precursor cells (OPCs) during development. Studies have shown that this gene is required for the generation of oligodendrocytes, as *Olig2* knockout mice lack oligodendrocytes [9]. Therefore, the forced expression of Olig2 is a promising method to promote the oligodendrogenic fate of cells. By directly targeting Olig2, this approach can more specifically and effectively promote oligodendrogenic differentiation compared to the use of small molecules, which may not be able to fully replicate the complex regulatory mechanisms that occur during natural oligodendrocyte generation. Notably, forced Olig2 expression has successfully resulted in the conversion of murine, rat and human fibroblasts into OPCs when combined with other oligodendrogenic transcription factors [10,11]. Moreover, Olig2 alone has been shown to be sufficient to generate OPCs from neural stem cells [12]. In the present study, we built on this literature by developing inducible oNPCs (ioNPCs) that could be tailored based on different timelines of doxycycline treatment. We further aimed to use RNA sequencing with gene ontology (GO) and gene set enrichment analysis (GSEA) in order to characterize and compare the transcriptome of the ioNPCs to that of untreated NPCs. Our goal was to assess the therapeutic relevance of the ioNPCs for the potential treatment of oligodendrocyte-related neurological diseases.

## 2. Materials and Methods

### 2.1. Lentivirus Packaging

293T cells were seeded in a T-75 flask at a density of 1 × 10^6^ cells per flask in 15 mL of DMEM culture medium containing 10% fetal bovine serum and 1% Penicillin/Streptomycin. A plasmid transfection mixture was prepared by mixing the tet-O-FUW-Olig2 plasmid (1 µg; Addgene #45844), psPAX2 plasmid (1 µg) and pMD2.G plasmid (0.5 µg) in 1 mL of Opti-MEM medium [10]. A Lipofectamine transfection reagent was prepared by mixing 3 µL of Lipofectamine 3000 (Invitrogen, Cat. #18324012) with 1 mL of Opti-MEM medium (Gibco, Cat. #319850070. The plasmid transfection mixture was added to the Lipofectamine transfection reagent and incubated at room temperature for 20 min. The Lipofectamine and plasmid transfection mixture was then added to the 293T cell culture media, and the cells were incubated at 37 °C, 5% CO_2_ for 48 h. The culture medium containing the virus was collected and centrifuged at 3000× *g* for 10 min to remove any debris. The supernatant was transferred to ultracentrifuge tubes and centrifuged at 27,000× *g* for 2 h at 4 °C to pellet the virus. The virus pellet was resuspended in 1 mL of phosphate-buffered saline (PBS; Wisent, Cat. #311-012-CL), filtered through a 0.45 um filter to remove any remaining debris and centrifuged at 27,000× *g* for 2 h at 4 °C to pellet the virus again. The virus pellet was resuspended in a small volume of PBS, and the virus concentration was measured using a spectrophotometer at 260 nm. The virus was stored at −80 °C until ready to use.

### 2.2. ioNPC Generation

The BC1 line of human-induced pluripotent stem cells (hiPSCs) (provided by the Centre for Commercialization of Regenerative Medicine (CCRM) in Toronto, ON, Canada) was differentiated to NPCs using dual SMAD inhibition, and the cells were maintained at a low passage [13,14]. After the differentiation of the hiPSCs into NPCs, we transfected the cells with piggybac vectors that express the transcriptional activator protein rtTA under the control of an EF1a promoter. Additionally, the plasmid also includes GFP expressed under the control of an IRES element downstream of the *rtTA* gene, allowing for the visualization of cells in which the rtTA protein is active. The NPCs were then infected with the Olig2 lentivirus, and the infected cells were purified by generating neurospheres in clonal density. The cells were subsequently expanded and treated with doxycycline (Bio Basic, Cat. #DB0889) at a concentration of 1 μg/mL for 3, 7 or 10 days to activate the tet-ON promoter and the associated Olig2 transcription factor. Untreated cells were used as a control.

### 2.3. qRT-PCR

The ioNPCs that were treated with doxycycline for 3, 7 or 10 days, as well as the untreated cells, were collected into a tube containing Buffer RL with β-mercapthenol. Small-molecule-treated oNPCs were also collected. The samples were then processed according to the manufacturer’s instructions using a Total RNA Purification Kit (Norgen Biotek, Cat. #17200), which involved adding a lysis buffer to the samples and purifying the RNA using a spin column. To synthesize cDNA, purified RNA was added to a reaction tube along with the appropriate amount of SuperScript First-Strand Synthesis System for RT-PCR (Thermo Fisher Scientific, Cat. #11904018), and the manufacturer’s instructions were followed to complete the cDNA synthesis reaction. Specific regions of the cDNA were amplified using RT-PCR by setting up the reaction with the GeneAmp PCR System 9700 and adding the appropriate amount of cDNA, forward and reverse primers, and TaqMan probes (detailed information regarding the TaqMan probes can be found in Table S1). The polymerase was activated, and the DNA was denatured by heating the reaction mixture to 94 °C for 2 min. Then, 35 cycles of RT-PCR were performed using the following conditions: 94 °C for 30 s, 58 °C for 30 s and 72 °C for 60 s. The values were normalized to the *GAPDH* housekeeping gene.

### 2.4. Immunocytochemistry

To study the differentiation potential of the cells in vitro, the control untreated NPCs or the ioNPCs that had been treated with doxycycline for 7 days were dissociated into single cells and plated on polyornithine/Laminin-coated cover glasses (24 well plates: 4 × 10^3^ cells/well). The cells were grown in a neurobasal medium (Thermo Fisher Scientific, Cat. #21103049) supplemented with N2 (Thermo Fisher Scientific, Cat. #17502048), B27 (Thermo Fisher Scientific, Cat. #17504044), 0.1% fetal bovine serum, 10 μM Forskolin (Stem Cell Technologies, Cat. #72112) and Glutamax (Thermo Fisher Scientific, Cat. #21103049) for an additional 10 days. The cells were then fixed for 20 min with 4% paraformaldehyde (BioShop, Cat. #PAR070) in PBS and 40% sucrose (BioShop, Cat. #SUC507) at room temperature. Following fixation, the cells were permeabilized in 0.1% Triton X-100 (BioShop, Cat. #TRX7777) and 0.1% sodium citrate (Sigma-Aldrich, Cat. #S4641) in PBS for 5 min and then placed in a blocking buffer (5% BSA) for 1 h. Primary antibodies were diluted in the blocking buffer solution and incubated with the cells overnight at 4 °C. Following extensive washing, the samples were incubated with 4′,6-diamidino-2-phenylindole (DAPI, 1:1000, Invitrogen, Cat. #D1306) and fluorophore-conjugated secondary antibodies for 1 h. To quantify, three wells stained for NESTIN (rabbit IgG, 1:500, Millipore, Cat. #AB5922), βⅢ TUBULIN (mouse IgG2b, 1:1000, Sigma Aldrich, Cat. #T8660), glial fibrillary acidic protein (GFAP; mouse IgG1, 1:1000, Millipore, Cat. #MAB360) and O1 (mouse IgM, 1:500, Millipore, Cat. #MAB344) were counted within the field of view in five areas within each well. The percentage of differentiated cells was calculated relative to the total number of cells in each field of view.

### 2.5. RNA Sequencing

Three biological replicates of rRNA-depleted stranded libraries were prepared for the control NPCs and the ioNPCs that were treated with doxycycline for 7 days, and they were sequenced independently. The prepared libraries were multiplexed and paired-end 75 bp sequencing was performed on the multiplexed libraries using an Illumina NextSeq 550 sequencer, aiming to obtain 2–3 million sequencing reads for each sample. The raw sequence reads were aligned to the UCSC human reference genome (hg19) using TopHat with default parameters, and HTSeq was used to count the number of mapping reads using the UCSC hg19 reference genome annotation. The Illumina TruSeq adapters were trimmed from the FASTQ files using Trim Galore (version 0.4.4), and the FASTQ files were aligned to the GRCh38 reference genome using STAR software (version 2.6.0c) with the specified options. StringTie (version 1.3.3b) was used to quantify TPM for the genes in the GRCh38 annotation.

### 2.6. Differential Gene Expression Analysis

Differential gene expression (DGE) analysis was performed on the TPM data using the Limma package in R (version R-4.2.2). The input data consisted of a matrix of TPM values, with the genes as rows and the samples as columns. The data were log-transformed using log2(TPM + 1) to stabilize the variance and increase the dynamic range of the data. The voom transformation was applied to the data to account for the differences in the sequencing depth. Two groups, the NPC controls and the ioNPCs following 7 days of doxycycline treatment, were defined with 3 samples in each group. A design matrix was created using the Groups variable, and a linear model was fit to the data using the lmFit function. A contrast analysis was performed using the makeContrasts function to compare the two groups, and a moderated *t*-test was performed using the eBayes function. *p*-values and log fold-changes were extracted using the topTable function with the false discovery rate (FDR) adjustment. To visualize the sample similarities, a multidimensional scaling (MDS) plot was created using the plotMDS function from the Limma package. Principal component analysis (PCA), volcano plot generation and hierarchal clustering were performed in R.

### 2.7. Gene Ontology Analysis

The process of identifying significant differentially expressed genes began by filtering out those that did not meet the criteria of p.adj < 0.05. The gene IDs that were found to be upregulated or downregulated were then separately uploaded to the ShinyGO 0.77 platform, with humans as the organism of interest [15]. To ensure accuracy, the false discovery rate cutoff was set at 0.05 and the number of pathways analyzed was limited to 20, with a minimum pathway size of 2 and a maximum of 2000. The pathway databases used included GO Biological Process, GO Cellular Compartment and GO Molecular Function. The results were then downloaded, and the top 10 pathways with the highest fold enrichment were visualized using the ggplot2 R package.

### 2.8. Assessment of Cell Lineage Gene Programs Using Gene Set Enrichment Analysis

To understand the cellular components of the ioNPCs following 7 days of doxycycline treatment and to compare them to those of the control NPCs, we utilized a custom gene set enrichment method through GSEA. To assess the gene programs in the bulk RNA-seq samples, we compiled a reference panel of 42 gene signatures from published scRNA-seq atlases [16,17,18,19,20,21] (Table S2), covering various stages of NPC and OPC differentiation. The GSEA analysis was carried out using these signatures as input and applied to our bulk RNA-seq data. The normalized enrichment scores (NES) were calculated by normalizing the enrichment to the average enrichment of 10,000 random gene samples. Significantly enriched results were defined as having nominal *p* values < 0.25.

## 3. Results

### 3.1. Morphological and Transcriptional Analyses of ioNPCs Reveals Progressive Oligodendrogenic Lineage Development following Doxycycline Administration

**Figure 1:**
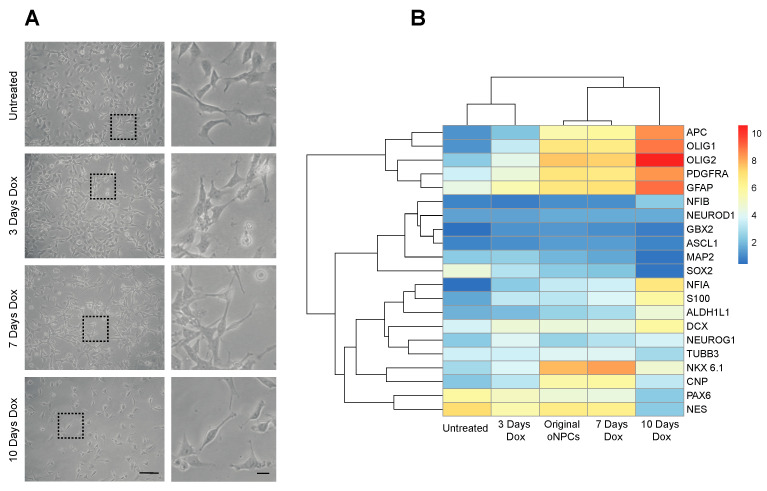
Morphological and transcriptional changes in the ioNPCs over the course of doxycycline treatment. (**A**) Microscopic images of untreated NPCs and ioNPCs over the course of 3, 7 and 10 days of doxycycline treatment. The dotted boxes can be seen enlarged to the right. Scale bars = 50 µm left and 5 µm right. (**B**) Heatmap representing changes in the expression of selected neural lineage genes analyzed using qRT-PCR. The heatmap shows the relative expression levels of the genes at different time points of doxycycline treatment, with red indicating upregulation and blue indicating downregulation.

First, we generated NPCs from hiPSCs using a dual SMAD inhibition method and then infected the cells with a lentivirus expressing the Olig2 transcription factor under the control of a tet-ON promoter. The infected cells were subsequently purified and left untreated or treated with doxycycline for 3, 7 or 10 days to induce the expression of Olig2. Our results demonstrate that the doxycycline treatment led to distinct changes in the morphology of the ioNPCs, as evidenced by a shift from a polygonal cell shape with multipolar processes to a ramified morphology characterized by a complex network of processes surrounding the cell body and sometimes presenting a bipolar shape. These morphological changes were observed consistently across multiple experiments and provide visual evidence of the impact of Olig2 expression on the cellular architecture of ioNPCs (Figure 1A). Furthermore, the transcriptional changes in the cells were monitored in the untreated and treated cells (Figure 1B). Following 3 days of doxycycline treatment, the ioNPCs had a transcriptional profile similar to that of the untreated control NPCs, with the maintenance of NPC markers, such as *PAX6* and *NES*. Further administration of doxycycline for a total of 7 days resulted in a transcriptional profile very similar to that of our original oNPCs. Several genes related to the oligodendrogenic lineage became upregulated, including *APC*, *OLIG1*, *OLIG2*, *PDGFRA*, *NKX6.1* and *CNP*. At this stage, the cells maintained the expression of *PAX6* and *NES*. At 10 days of treatment, the expression of oligodendrogenic genes further increased, whereas *PAX6* and *NES* expression decreased. The transcriptional profile suggests that these cells may correspond to OPCs.

### 3.2. In Vitro Differentiation Analysis of the Doxycycline-Treated ioNPCs Reveals a Bias along the Oligodendrogenic Lineage

**Figure 2:**
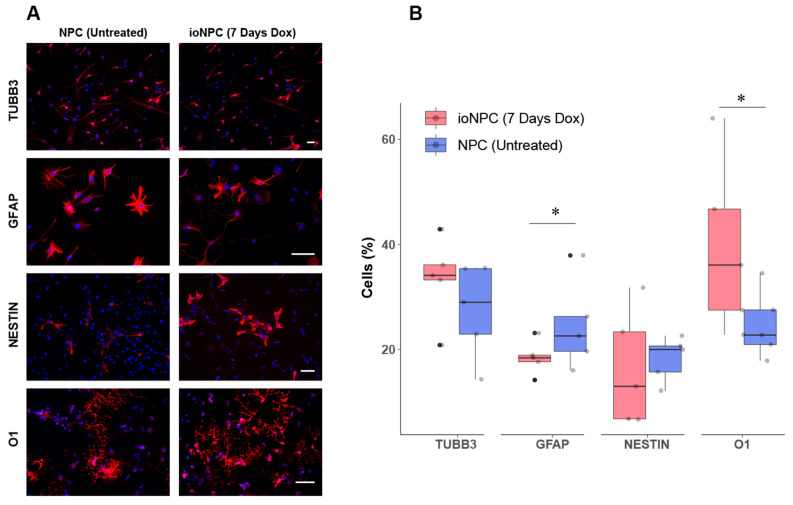
Differentiation profile of untreated control NPCs in comparison to ioNPCs that were treated with doxycycline for 7 days. (**A**) Fluorescent images of TUBB3, GFAP, NESTIN and O1 (red) and DAPI (blue) staining after 10 days of differentiation in 0.1% fetal bovine serum. Scale bar = 20 µm. (**B**) Graph showing the percentage of neurons (TUBB3), astrocytes (GFAP), undifferentiated cells (NESTIN) and oligodendrocytes (O1) following 10 days of differentiation in 0.1% fetal bovine serum. Gray points refer to each individual datapoint and black points represent outliers, which are outside of the range of the “whiskers” of the box plot. Error bars represent SEM, *n* = 3, and statistical significance was determined using Student’s *t*-test (* *p* < 0.05).

To confirm that the ioNPCs were successfully biased along the oligodendrogenic lineage at 7 days, we differentiated the cells and the NPC controls in vitro (Figure 2A) and quantified the percent differentiation (Figure 2B). The ioNPCs gave rise to significantly more O1-positive oligodendrocytes following 7 days of doxycycline treatment than the unbiased NPCs that were not treated with doxycycline (ioNPC: 39.44 ± 16.5%, NPC: 24.73 ± 6.5%). The percent differentiation to TUBB3-positive neurons was 33.45 ± 8% in the ioNPCs and 27.43 ± 9% in the NPCs, whereas the percentage of astrocytes (GFAP) decreased from 24.5 ± 8.4% to 18.46 ± 3.2%. The percentage of undifferentiated NESTIN-positive cells did not change significantly (ioNPC: 16.32 ± 11%, NPC: 18.23 ± 4.23%). These results suggest that the doxycycline-induced expression of Olig2 in ioNPCs promotes the differentiation of ioNPCs toward the oligodendrocyte lineage, as indicated by the increased percentage of O1-positive cells (Figure 2B).

### 3.3. RNA Sequencing Analysis of the ioNPCs Reveals Differentially Expressed Genes Linked to the Commitment of the Oligodendrogenic Lineage

**Figure 3:**
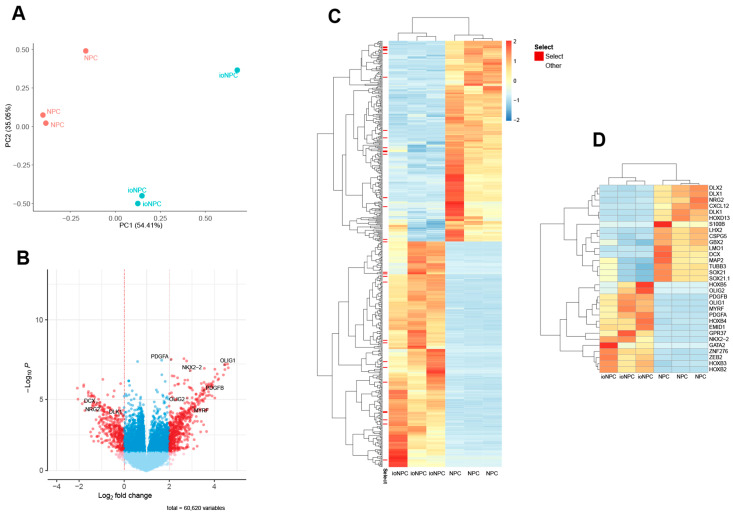
RNA-seq analysis of the ioNPCs following 7 days of doxycycline treatment and control NPCs with three replicates each. (**A**) PCA plot showing the clustering of the ioNPC and NPC samples based on the DEGs. (**B**) Volcano plot showing the DEGs between the ioNPCs and NPCs, with the significant DEGs represented by red dots (FDR < 0.05). (**C**) Heatmap of all DEGs. The color scale represents the log2 fold-change in the expression of each gene in the ioNPCs in comparison to that in the NPCs, with red indicating high expression and blue indicating low expression. (**D**) Heatmap of selected DEGs. Genes related to the oligodendrocyte lineage, including *PDGFB*, *OLIG1*, *MYRF*, *PDGFA* and *NKX2.2* were upregulated in the ioNPCs, whereas neuronal genes, such as *DCX*, *MAP2* and *TUBB3*, as well as the astrocyte gene, *S100B*, were preferentially expressed in the NPC controls. The color scale represents the log2 fold-change in gene expression in the ioNPCs compared to that in the NPCs, with high expression shown in red and low expression shown in blue.

The whole-genome expression profiles of the ioNPCs were analyzed and compared to those of the control, untreated NPCs. To evaluate the consistency and variance of the six samples, PCA based on all gene transcripts and hierarchical clustering based on all the differentially expressed genes (DEGs) were performed. The PCA results showed that the cell lines clustered together according to cell type, indicating that the inter-type cell line differences were maintained with respect to their biasing. Furthermore, the PCA plot demonstrated that the ioNPC samples were distinct from the control NPC samples, highlighting the effectiveness of the doxycycline treatment in inducing the oligodendrogenic fate of NPCs (Figure 3A). The RNA-seq analysis revealed a total of 521 significant DEGs, with 275 genes being upregulated and 246 genes being downregulated in the ioNPCs compared to those in the control NPCs (|log2 FC| ≥ 1 and FDR < 0.01; Figure 3B,C). Several genes related to the oligodendrocyte lineage, including *PDGFB*, *OLIG1*, *MYRF*, *PDGFA* and *NKX2.2*, were upregulated in the ioNPCs. On the contrary, neuronal genes, such as *DCX*, *MAP2* and *TUBB3*, as well as the astrocyte gene, *S100B*, were preferentially expressed in the NPC controls (Figure 3D).

### 3.4. Gene Ontology Analysis Reveals That the Upregulated Genes in the ioNPCs Correspond to Pathways Related to Oligodendrocyte Development

**Figure 4:**
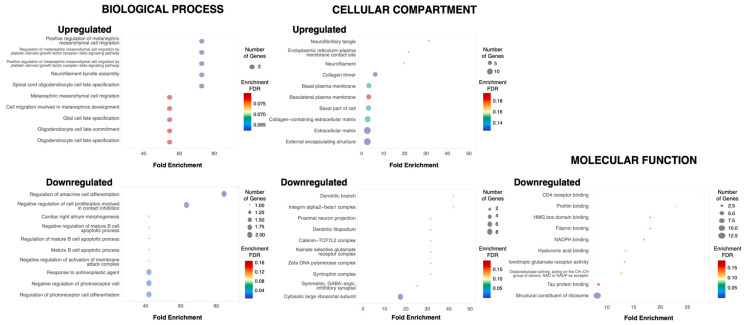
GO analysis of the DEGs. Summary of the upregulated and downregulated biological processes, cellular compartments, and molecular functions in the ioNPCs following 7 days of doxycycline treatment relative to those in the control NPCs. The circle diameter correlates with the number of genes, and the color is indicative of the enrichment FDR. No molecular functions were identified amongst the upregulated genes.

To gain insights into the potential functions of the DEGs, we performed functional annotation using the ShinyGO bioinformatics tool. The GO analysis revealed the enrichment of GO terms related to oligodendrocyte development, including spinal cord oligodendrocyte fate specification, oligodendrocyte cell fate specification, oligodendrocyte cell fate commitment and glial cell fate specification amongst the upregulated genes. Terms such as HMG box domain binding, ionotropic glutamate receptor activity and cell communication by chemical coupling were identified amongst the downregulated genes (Figure 4). This is consistent with the hypothesis that the induction of Olig2 expression promotes the oligodendrogenic fate of NPCs and leads to oligodendrocyte differentiation.

### 3.5. ioNPCs Exhibit Enrichment in the Gene Expression Signatures of Distinct Cells along the Oligodendrogenic Lineage

**Figure 5:**
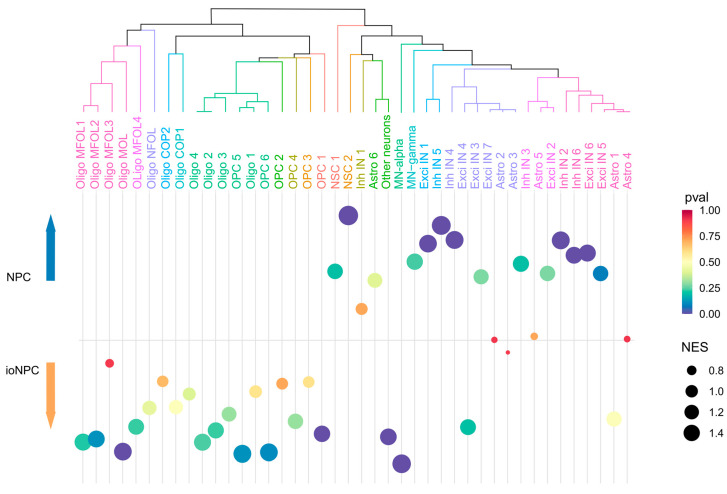
GSEA comparison of the control NPCs and 7-day doxycycline-treated ioNPCs in relation to published scRNA-seq atlases of different stages of NPC differentiation. Circle diameters indicate the normalized enrichment scores (NES), whereas the x-axis represents the cell types. Cell types that are closer to the top or bottom of the graph are more enriched in the control NPCs or in the ioNPCs, respectively. The dendrogram was generated based on the similarities of the gene expression signature of each cell subtype.

Next, we used cell-type-specific gene signatures from published scRNA-seq atlases as input for GSEA to characterize the cell identity programs that were enriched or depleted in the NPCs and ioNPCs (Figure 5) [16,17,18,19,20,21]. The ioNPCs were demonstrated to be enriched for oligodendrocyte precursor cells (OPCs) and oligodendrocytes at different stages of their development. Additionally, motor neurons (MNs) were enriched in the ioNPCs. In contrast, the control NPCs, which were not treated with doxycycline and, therefore, not biased toward an oligodendrogenic path, were enriched for different subtypes of inhibitory and excitatory interneurons (INs). However, it is important to note that GSEA measures the enrichment of the expression signatures associated with each cell type or subtype in a sample but not the proportion of different cell types in a population of cells. Therefore, it is important to be cautious when interpreting the results and to use other methods or techniques, such as scRNA-seq or flow cytometry, to validate the results for a more accurate measurement of the proportion of different cell types in the population.

## 4. Discussion

Cell therapies are a promising regenerative strategy for a variety of CNS disorders, including those associated with injury, a loss of neural cells and demyelination. To date, there has been a rigorous search to identify the optimal cell type that can target these conditions [4]. However, it is becoming increasingly apparent that there is a great amount of heterogeneity across different CNS disorders, arising from various injury severities, locations, cell types affected and timelines. Therefore, distinct stem cell therapies may be suitable for different circumstances. For example, OPCs are unipotent cells that have been commonly investigated for the purpose of promoting remyelination. A variety of small-molecule and transcription-factor-mediated methods have been used in order to derive OPCs, which express *OLIG1*, *OLIG2*, *PDGFRA*, *SOX10* and *NKX2.2* and can differentiate into O1-, O4-, MBP- and MOG-positive oligodendrocytes [22]. However, OPCs alone may not be sufficient to address the neuronal pathology that often accompanies oligodendrocyte loss in many neurological diseases. Injuries such as brain or spinal cord injuries lead to the widespread death of several cell lineages, including neurons [23]. Therefore, the transplantation of OPCs may not be sufficient if there are not enough endogenous spared axons that the OPC-derived oligodendrocytes can myelinate. Meanwhile, neuronal replacement may not be necessary in other conditions, such as multiple sclerosis, in which oligodendrocytes are particularly damaged [24]. Novel treatment options will need to reflect this diversity to be successful in a clinical setting. Efforts are now being invested into the identification of biomarkers, which would be able to help to stratify patients such that they can be assigned to the optimal treatment paradigm [25]. For example, sophisticated imaging techniques, such as diffusion tensor imaging or magnetic resonance imaging, can be applied to distinguish between patients who have a greater extent of white matter damage and demyelination vs. those that do not [26,27,28]. These clinical assessments can help to determine which terminal cell fates would be the most therapeutically relevant in a patient-specific manner. In this regard, our ioNPCs introduce an ideal treatment option for a variety of patients, as this is a single source of cells in which the extent of oligodendrogenic biasing can be tailored and personalized prior to transplantation depending on the amount of doxycycline administration. In our results, we found that increasing doxycycline treatments contributed to sequential changes in the gene expression of the cells. Following 3 days of doxycycline administration, the cells closely resembled NPCs, whereas by 10 days, the ioNPCs were enriched for oligodendrogenic genes, including *APC*, *OLIG1*, *OLIG2* and *PDGFRA*, with a simultaneous loss of neural progenitor markers, thus suggesting that they corresponded to OPCs at this stage. Seven days of treatment led to the generation of cells that were intermediate between the NPC and OPC stage and that differentiated into a greater proportion of O1-positive oligodendrocytes while remaining tripotent. As such, this Olig2-inducible system would allow clinicians to customize the extent of oligodendrogenic biasing toward the individual’s needs based on clinical assessments of their white matter damage.

Following injury and demyelination, a plethora of inhibitory signals and myelin debris related to the Notch, Wnt, Rho and PKC signaling pathways, amongst others, are released into the microenvironment. These signals ultimately skew the differentiation of progenitor cells and prevent the generation of new oligodendrocytes [5,29,30,31,32]. We aimed to address this inhibitory microenvironment by pre-conditioning NPCs along the oligodendrogenic lineage prior to transplantation. However, these pre-conditioned cells may remain susceptible to the influences of endogenous signaling cascades following transplantation. Therefore, the doxycycline system is particularly advantageous, as it permits the further in vivo manipulation of cell fate after the cells have been transplanted. Doxycycline is a tetracycline antibiotic derivative that is readily used in humans, making it relevant for in vivo application. It has been shown to cross the blood–brain barrier, thus enabling it to act on cells that have been grafted into the CNS [33,34,35]. Therefore, in vivo doxycycline treatment can alter cell fate to overcome the complex signaling cascades within the host microenvironment. Furthermore, the doses of doxycycline could be changed over time as the injury progresses in order to reflect the dynamic changes that occur as the damage progresses. As such, this inducible system can help to ensure the optimal differentiation of transplanted cells.

In recent years, advances in transcriptional techniques have allowed for the identification of distinct cell types along the trajectory of the oligodendrogenic lineage. Furthermore, it has been found that the heterogeneity of these cells is altered in demyelinating disorders, such as multiple sclerosis and spinal cord injuries [36,37]. We therefore characterized the identity of our cells using GSEA and found that a number of these cell types were represented in the ioNPCs. This included differentiation-committed oligodendrocyte precursors (COPs), which correspond to a fate that is intermediate between NPCs and OPCs [38,39]. Additionally, newly formed oligodendrocytes (NFOLs), which arise at the early stages of differentiation, as well as myelin forming oligodendrocytes (MFOLs), which express myelin-related genes, were also represented, suggesting that the ioNPCs express the genes that are necessary for maturation and myelin formation [38]. As such, our cells correspond to a variety of cells along the oligodendrogenic lineage. Nonetheless, there are also regional differences that exist across oligodendrogenic cells found in different areas of the CNS [40,41]. In this regard, we believe that using Olig2 induction is particularly relevant for the generation of cells that are suitable for a variety of demyelinating conditions, as Olig2 is ubiquitously expressed in oligodendrogenic lineage cells throughout the CNS [42,43,44,45].

Interestingly, our GSEA results also found that the ioNPCs corresponded to motor neurons. This is consistent with the fact that oligodendrocytes and motor neurons both originate from the ventral pMN progenitor domain in the spinal cord during development [9,43,46]. Specifically, sonic hedgehog signaling induces Olig2 expression, which initially leads to the production of motor neurons, followed by a shift toward the generation of ventral oligodendrocytes. This fate switch is regulated by interactions with other transcription factors, as well as the phosphorylation and dephosphorylation of Olig2 [47,48,49]. As such, Olig2 plays an essential role in motor neuron production, in addition to oligodendrocyte generation. Forced Olig2 expression in pluripotent cells has furthermore been reported to result in the generation of motor neurons [50]. Overall, this suggests that inducing Olig2 expression in the ioNPCs may bias the ioNPC-derived neurons toward a motor neuron cell fate upon differentiation, which may be particularly relevant in the context of spinal cord injury, in which motor neurons residing in the corticospinal tract are often damaged [51]. As such, further analysis will be required to characterize the neuronal subtypes derived from our ioNPCs. Furthermore, the ioNPCs may give rise to other regenerative effects beyond oligodendrocyte and neuron replacement. Notably, progenitor cells are known to secrete neurotrophic factors, including brain-derived neurotrophic factor (BDNF) and glial-derived neurotrophic factor (GDNF), which are known to support axonal growth and sparing, promote synaptogenesis, reduce astrogliosis, enhance cell proliferation and reduce inflammation [4,52,53,54,55,56]. Interestingly, these neurotrophic factors were not differentially expressed between our NPCs and ioNPCs, which may indicate that both cell sources can give rise to neurotrophic effects. Nonetheless, additional studies will be required to confirm this and to assess the various regenerative effects of the ioNPCs.

## 5. Conclusions

In the present paper, we developed an oligodendrogenic cell source that is suitable for a variety of CNS disorders associated with white matter injury or degeneration. We found that the incremental induction of Olig2 expression can give rise to cells with distinct morphological and transcriptional profiles. Additionally, we showed that 7 days of doxycycline treatment was enough to increase the efficiency of oligodendrocyte differentiation while maintaining the tripotent potential of the cells. RNA sequencing analyses further corroborated the pro-oligodendrogenic fate of the cells. As such, this is a promising cell source for in vivo evaluations in the future.

## Data Availability

Data are available from the corresponding authors upon reasonable request.

## Supplementary Materials

The following supporting information can be downloaded at https://www.mdpi.com/article/10.3390/cells12091252/s1, Table S1: The list of TaqMan probes; Table S2: Reference panel of 42 gene signatures from published scRNA-seq atlases.
